# The ReSPonD trial - rivastigmine to stabilise gait in Parkinson’s disease a phase II, randomised, double blind, placebo controlled trial to evaluate the effect of rivastigmine on gait in patients with Parkinson’s disease who have fallen

**DOI:** 10.1186/1471-2377-13-188

**Published:** 2013-12-03

**Authors:** Emily J Henderson, Stephen R Lord, Jacqueline CT Close, Andrew D Lawrence, Alan Whone, Yoav Ben-Shlomo

**Affiliations:** 1 Department of Social and Community Medicine, University of Bristol, Canynge Hall, 39 Whatley Road, BS8 2PS Bristol, UK; 2 Neuroscience Research Australia, University of New South Wales, Sydney, Australia; 3 Prince of Wales Clinical School, Department of Geriatric Medicine, Sydney, NSW, Australia; 4 School of Psychology, Cardiff University, Cardiff, UK; 5 Department of Neurology, Institute of Clinical Neurosciences, University of Bristol, Bristol, UK; 6 North Bristol NHS Trust, Bristol, UK

**Keywords:** Randomised control trial, Parkinson’s disease, Accidental Falls, Freezing of gait, Intervention, Gait analysis, Acetylcholinesterase, Cognitive, Attention, Dual-tasking

## Abstract

**Background:**

Gait impairment is common in people with Parkinson’s disease. There is a lack of effective interventions to target this debilitating complication and therefore a need to identify new therapeutic options. An underlying cholinergic deficit contributes to both the gait and cognitive dysfunction seen in Parkinson’s disease. The combined impact of both impairments can be assessed in gait tasks performed with concomitant cognitive tasks. The aim of this trial is to evaluate the impact of a cholinesterase inhibitor on cognitive function and gait performance in people with established Parkinson’s disease.

**Methods/design:**

This is a single centre, double-blind, randomised placebo-controlled trial in 130 people with Hoehn and Yahr stage 2–3 idiopathic Parkinson’s disease who have fallen in the past year. Participants will be randomised to two groups, receiving either rivastigmine capsules or identical placebo capsules for 8 months. Assessment will be undertaken at baseline and at the end of medication prescription (i.e. 8 months) with participants remaining enrolled in the trial for a further 4 months to monitor for falls and adverse events. The primary outcome is step time variability, assessed with and without the addition of concurrent cognitive tasks. Secondary outcomes will include other gait parameters, sensorimotor and balance performances, cognitive indices, falls and fall related injury, fear of falling, Parkinson’s symptoms and data pertaining to possible harms.

**Discussion:**

This randomised controlled trial will examine the effect of cholinesterase inhibitor therapy on gait, balance and falls in Parkinson’s disease. If effective, it would offer a new therapeutic option to ameliorating gait and cognitive deficits in a population at high risk of falls.

**Trial registration:**

ISRCTN19880883, UTN U1111-1124-0244.

## Background

Falls are a common and devastating complication of Parkinson’s disease (PD). Whilst tremor, akinesia and rigidity are early motor manifestations, postural instability tends to emerge as the disease progresses. Postural instability, coupled with gait dysfunction, is a major determinant of disability [1] with mobility and walking limitation cited by patients as the worst aspect of the disease [2]. Falls are a cause of significant morbidity. The consequences include fractures and injuries [3], fear of falling [4], nursing home and hospital admission [5] carer strain [6] and increased mortality [7]. Gait impairment, postural instability and falls are common complications of PD and may have a greater impact on quality of life than motor complications and dyskinesia [8]. There is currently a lack of pharmacological options of proven efficacy for addressing falls, postural instability, freezing of gait and festination in this high risk population.

The burden of disease complications will inevitably increase as the number of people with Parkinson’s disease in the most populous nations is expected to double from 2005 to 2030 [9]. A meta-analysis of prospective studies exploring falls in people with Parkinson’s disease demonstrated that 46% of individuals fall at least once over a period of three months [10]. Twenty years from diagnosis, 87% of people with Parkinson’s disease in the Sydney cohort had fallen with 35% sustaining a fracture [11]. The peak risk of falls corresponds with Hoehn and Yahr stage three [12] where people are still mobile but have reduced stability.

Despite increasing recognition of the non-motor features of the disease, the mainstay of pharmacological therapy remains dopaminergic medications. However, disorders of gait and balance in PD are not generally ameliorated by typical dopaminergic medication. During the maintenance and early complications phase of the disease, gait disturbances and end of dose freezing, typically respond to dopaminergic agents. As the condition progresses with associated motor complications, response to treatment becomes less predictable and freezing may develop in the “on” stage [13]. As Parkinson’s disease advances, “off” freezing becomes unpredictable despite optimisation of medical therapy and levodopa-induced dyskinesias and ‘on’ freezing further contribute to the risk of an individual falling.

Characterisation of the cholinergic neurochemical deficit that is thought to underlie gait disorders and postural instability has aided the search for potential therapeutic targets. The predominantly cholinergic pedunculopontine nucleus (PPN) provides input into the basal ganglia and cortex and undergoes degeneration in PD. The extent of degeneration is correlated with the degree of gait dysfunction [14]. Cortical and thalamic cholinergic loss, representing projections from the nucleus basalis of Meynert (nbM) and the PPN respectively, is significantly greater in people with PD and a history of falls compared to both those without falls and non-PD controls [15]. Similarly, the degree of cholinergic neuronal loss is negatively correlated with the Hoehn and Yahr score, which is driven by the progressive emergence of axial impairment [16].

Outside of a potential role in gait dysfunction, cholinergic loss manifests as an alteration in attention and cognition [17]. Furthermore mild cognitive impairment (MCI) is highly prevalent in people with PD and increases with age, duration and severity of PD and affects a range of cognitive domains [18]. The nbM is the main source of cholinergic projections to the cerebral cortex and degenerates in PD [19]. Greater cholinergic neuronal loss in the nbM is seen in people with Parkinson’s disease dementia (PDD) than in PD [20], and neuroimaging evidence of this cholinergic dysfunction is present early in the disease course [21].

The observed deficit in cholinergic function in Parkinson’s disease thus accounts, at least to some degree, for the dysfunction seen in both cognitive and gait domains. Historically, gait has been regarded as demanding little or no cognitive resources. The dual-task technique provides a means to examine the extent to which different activities demand attentional capacity, i.e. draw on common central processing resources. In dual-task experiments, a decrement in performance occurring when two tasks are performed simultaneously (relative to baseline performance on each single task) indicates that both tasks demand attention [22].

Dual-task paradigms have revealed important insights into the relationship between gait and cognition. Dual-task conditions augment gait impairments seen during normal walking in PD such as reduction in gait speed, stride length and increased stride-to-stride variability [23]. Furthermore, in PD, a ‘posture second’ strategy seems to be adopted whereby individuals are unable to appropriately prioritise the safety of their walking above other cognitive demands [24].

Despite evidence that cholinergic loss may influence both gait and cognitive changes, few have studied the effect of cholinergic augmentation on gait, balance and falls. A small trial of six individuals with Alzheimer’s disease (AD) versus eight controls with MCI examined the effect of a 4 month period of treatment with the acetylcholineseterase inhibitor donepezil. This demonstrated that both gait velocity and variability improved under single and dual-task conditions at follow-up [25]. Two further studies showed an improvement in stride time [26] and a reduction in falls with treatment [25,27]. A further RCT is currently underway examining these effects on a larger cohort of individuals with MCI [28]. In Parkinson’s disease, a small cross over trial of donepezil showed a reduction in fall rate, though this was driven primarily by reductions in only those with a history of very frequent falls [27].

The aim of this randomised controlled trial is to examine the effect of cholinesterase inhibitor therapy on gait and balance and fall risk in people with Parkinson’s disease with a history of one or more falls in the past year.

## Methods

### Design

**Figure 1:**
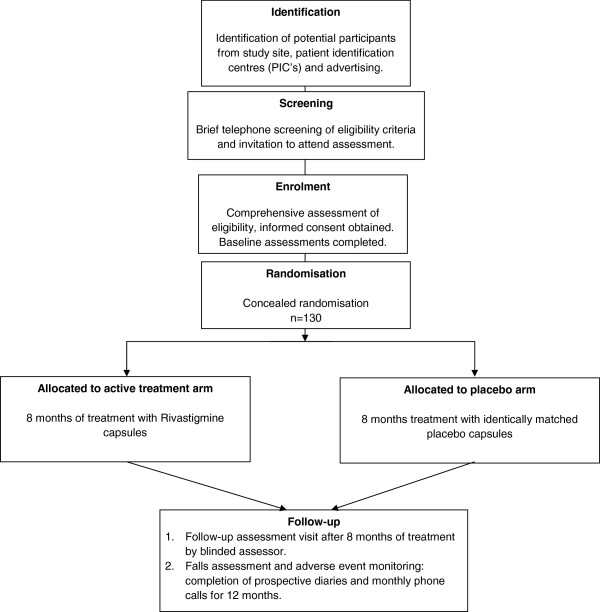
Trial flow diagram.

A placebo-controlled double blind, randomised clinical trial (RCT) using a parallel arm design (Figure 1).

### Participants and setting

Participants will be eligible if they meet the following inclusion criteria; a) have moderate Parkinson’s disease (Hoehn and Yahr stage 2–3), b) have fallen in the last year, c) are able to walk without aids for the distance of the walking protocol (approximately 18 metres), d) have been stable on anti-Parkinsonian medication for 2 weeks prior to enrolment and e) are able to give informed consent. Potential participants will be excluded if they: a) have any other cause contributing to their Parkinsonian features (vascular disease, multisystem atrophy (MSA), progressive supranuclear palsy (PSP), normal pressure hydrocephalus), b) have a known diagnosis of dementia, c) have other neurological, visual or orthopaedic problems that significantly interfere with balance or gait, d) have had previous or current treatment with a cholinesterase inhibitor or an absolute contraindication to cholinesterase inhibitor therapy, e) are unable to attend or comply with treatment or follow-up scheduling and f) are non-English speaking as cognitive tests will be performed in English.

Ethical approval was granted from the South West Research Ethics Committee on 28^th^ September 2011 and the Medicines and Healthcare Regulatory Agency (MHRA) on 18^th^ June 2012.

Participants will be identified from centres in the South West of England and South East Wales by Parkinson’s specialist clinicians, through the DeNDRoN (Dementias and Neurodegenerative Diseases Research Network) and via advertising through the Parkinson’s Disease-UK charity. Participants will also be identified through National Health Service (NHS) Trusts acting as Participant Identification Centres (PICs) predominantly in neighbouring regions of South West England. The trial is a single site study based in a NHS Trust hospital.

Participants will be sent or given an information pack that provides further details about the study and asked to return a reply slip indicating their interest in taking part. Interested participants will be screened by telephone and offered an appointment if they meet the inclusion criteria. Written informed consent will be obtained from all participants.

### Randomisation and blinding

After confirming eligibility and obtaining informed consent, participants will be randomised to one of two treatment arms. Both the assessors and participants will be blinded to the treatment allocation throughout the trial. Unblinding will occur once all assessments are complete and the database has been locked at the end of the trial. The randomisation sequence will be generated by Bristol Randomised Trials Collaboration (BRTC) clinical trials unit. We will use a minimisation algorithm with a random element to ensure that the two trial arms are similar on three key variables: age, cognitive impairment (using the Montreal Cognitive Assessment (MOCA)) and number of falls sustained in the past year. The list will be generated by a web based program which then issues a treatment pack number that is matched to a drug package held in the pharmacy, thereby ensuring concealment of allocation.

### Assessment procedures

Participants will visit the hospital on two occasions to undergo assessment, at baseline and after 32 weeks of treatment. The baseline visit will involve checking eligibility against the inclusion and exclusion criteria and administering questionnaires relating to sociodemographic and general health measures. Assessment of physical and cognitive function, a physical examination and recording of an electrocardiogram will also be undertaken. If eligible and willing to participate, informed consent will be obtained and participants will be randomised at this stage.

At both visits, assessments will include cognitive tests, analysis of fall risk, and gait and balance testing. Falls and adverse event data will be ascertained by monthly follow-up phone calls. Visit 2 will additionally involve ascertainment of osteoporosis risk factors and measurement of frailty status to reduce the assessment burden for visit 1. Table 1 illustrates the assessments performed at each visit and whether these are primary or secondary outcome measures.

**Table 1:** Assessments at baseline (BA) and at follow-up (FUA) indicating primary (1°) and secondary (2°) outcomes (O)

	**BA**	**FUA**	**O**
**Sociodemographics**			
Marital status, education, occupation, socioeconomic status, gender, ethnicity	✓	**x**	**x**
**General health and function**			
Past medical and Parkinson’s disease history, medication use, social history (place and type of residence, help with activities of daily living, smoking, alcohol use, use of walking and visual aids)	✓	✓	**x**
Osteoporosis risk factors are recorded according to the WHO Fracture Risk Assessment Tool (FRAX tool) [29] and the QFRACTURE [30] risk algorithm.	**x**	✓	**x**
Measurement of frailty status using the CSHA Clinical Frailty Scale [31] and the criteria proposed by Fried et al. [32] measuring weight loss, physical activity, grip strength, walk speed and self-reported fatigue.	**x**	✓	**x**
Self-reported physical activity measured using the Physical Activity Scale for the Elderly (PASE) [33]	**x**	✓	**x**
**Quality of life**			
ED-5D-5 L is widely used quality of life instrument which can be used for estimating QALYs for economic evaluations [34].	✓	✓	2°
**Cognitive assessments**			
Cognition will be assessed using the Montreal Cognitive Assessment (MOCA) [35]	✓	✓	2°
Cognitive Failures Questionnaire (CFQ) [36]	✓	✓	2°
Test Your Memory (TYM) [37]	✓	✓	**x**
Frontal Assessment Battery (FAB) [38]	✓	✓	2°
The 15 item Geriatric Depression Score will be used to measure mood [39].	✓	✓	2°
**Physical assessment**			
Physical examination and electrocardiogram	✓	**x**	**x**
Gait assessment using triaxial accelerometer with and without concurrent cognitive tasks	✓	✓	1°
Freezing of gait will be assessed with the specific gait task designed to elicit freezing as well as the Freezing of Gait Questionnaire [40].	✓	✓	2°
Coordinated stability – a measure of controlled leaning balance [41].	✓	✓	2°
Physiological Profile Assessment containing 5 validated measures of physiological function: visual contrast sensitivity, proprioception, quadriceps strength, simple reaction time, and postural sway while standing on a foam rubber mat with eyes open [42].	✓	✓	2°
Parkinson’s disease fall risk score comprised of 3 validated measures quadriceps strength, coordinated stability and freezing of gait [43].	✓	✓	2°
Parkinson’s disease severity will be measured using the Movement Disorder Society – Unified Parkinson’s Disease Rating Scale [44].	✓	✓	2°
**Falls, resultant injuries and adverse events**			
Falls, injuries and use of medical services will be collected by monthly phone calls and diaries.	✓	✓	2°
Adverse events will be screened for during monthly follow-up phone calls	✓	✓	2°

### Intervention

**Figure 2:**
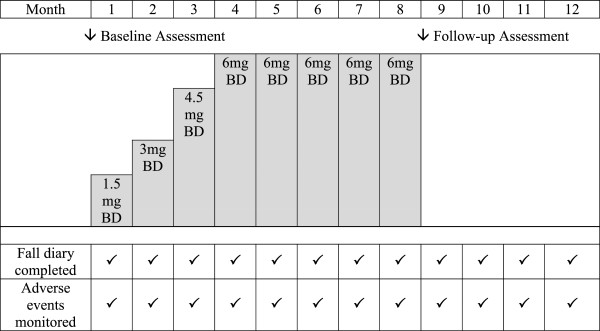
Schedule of assessment visits, medication titration and monitoring of adverse events and falls.

Participants will receive either rivastigmine or an identically matched placebo in treatment packs at visit one. They will be instructed to take one tablet twice a day. All participants will up-titrate the dose every four weeks (1.5 mg, 3 mg, 4.5 mg and 6 mg rivastigmine or matched placebo) over a 16 week period. This slower four week up-titration schedule has been adopted in other clinical trials on the basis of optimising tolerability [45,46]. The up-titration phase will be followed by a 16 week maintenance dose period after which time the medication will be stopped, yielding a total treatment period of 32 weeks (Figure 2). In the event of unacceptable side effects occurring, participants will be instructed to down-titrate to the last tolerated dose or stop the medication according to clinical judgement.

### Primary outcome measure - step time variability

The primary outcome measure will be step time variability assessed in three walking conditions: normal walking, (b) walking while performing a verbal fluency task and (c) walking while performing a verbal fluency switching task. Variability will be calculated according to current recommendations [47] as both the standard deviation (SD) and the coefficient of variation (CoV) (SD/mean x 100%). The SD for the three walking conditions will be reported as the primary outcome measure, though we hypothesise a priori that any observed differences will be magnified with increasing cognitive demands. Each condition is repeated three times for a total of nine walks. Gait will be assessed with an accelerometer, housed in a small, lightweight (5 g) container attached to the participant’s waist with an elasticated belt (Dynaport Hybrid system, McRoberts). Participants will be tested whilst in the ‘on’ medication state and will be instructed to walk at self-selected speeds over a 22 m obstacle-free level corridor, wearing their normal footwear and clothing. During the dual-task conditions, participants will not be given instructions as to which task to prioritise. The verbal fluency task is based on the controlled oral word association test (COWAT) whereby participants are asked to name as many words as they can beginning with a given letter of the alphabet. The switching dual-task is a more challenging version of COWAT. Participants are instructed to switch alternately between words beginning with two different letters of the alphabet. Letters are selected based on their frequency in the English dictionary and thus perceived difficulty. The three walking conditions will be randomly ordered to minimise practice effects. In the event of a failure on either the cognitive task or walking the participant will be prompted to continue. Cognitive task performance will be recorded using a lightweight non-obstructive dictaphone worn by participants.

### Secondary outcome measures

#### Additional gait measures

In addition to step time variability, we will assess the effects of the intervention on a number of other gait parameters: harmonic ratios, velocity (m/s), cadence (steps/min), step length (cm), stance time (sec), stance time variability, double limb support (% of gait cycle) and walk ratio (see Appendix 1 for more details).

Freezing of gait will be assessed by asking participants to stand from a chair, walk between two chairs placed 50 cm apart, turn 360° to the right, 540° to the left and then walk back the same way and return to the chair. The walks will be videotaped to classify the type and duration of any freezing episodes. Freezing of gait will be also measured using the FOG questionnaire [40].

#### Controlled leaning balance

Controlled leaning balance will be assessed with the coordinated stability test which requires participants to accurately adjust their position in a steady and coordinated manner when their centre of mass is near the limits of their base of support [41].

#### Fall risk indices

Fall risk will be assessed with two validated mea-sures - the short-form Physiological Profile Assessment (PPA) [42] and the Parkinson’s disease fall risk index (PDFRI) [48].

The PPA consists of five measures of sensorimotor performance including (1) visual contrast sensitivity, (2) proprioception, (3) quadriceps muscle strength, (4) hand reaction time and (5) postural sway. Visual contrast sensitivity is assessed using the Melbourne Edge Test. The chart has 20 circular patches containing edges with reducing contrast with variable orientation as the identifying feature. Correct identification of the orientation of the edge on the patches provides a measure of contrast sensitivity in decibel units, where 1 dB = 10 log_10_ contrast. Proprioception is measured using a lower-limb matching test, where difference in matching the great toes in degrees is recorded using a vertical clear acrylic sheet inscribed with a protractor and placed between the legs. Quadriceps muscle strength (isometric) in kilograms is examined in the dominant leg using a spring gauge while participants are seated with the hip and the knee joint at 90° of flexion. Simple reaction time in milliseconds is assessed using a light as a stimuli and a finger-depression of a switch as the response. Postural sway (total sway path in millimeters) is assessed using a swaymeter that measures the displacement of the body at waist level while participants stand for 30 seconds on a foam rubber mat with eyes open. The five PPA components are weighted to compute a composite PPA fall risk score which has been shown to prospectively discriminate between older fallers and non-fallers with an accuracy up to 75% [42].

The complementary PDFRI is calculated from an algorithm comprising measures of gait freezing, abnormal posture, executive functioning, coordinated stability and lower limb weakness. In a study of 113 participants with PD this index discriminated between fallers and non-fallers with a sensitivity of 77% and a specificity of 82% [48].

#### Cognitive assessment, mood and quality of life

Cognition will be measured using the Montreal Cognitive Assessment (MOCA) [49], Cognitive Failures Questionnaire (CFQ) [36], Test Your Memory (TYM) tool [37], Frontal Assessment Battery (FAB [38]), and verbal fluency performance assessed whilst seated using the COWAT (described above). Fear of falling will be quantified using the short version of the Iconographical Falls Efficacy Scale (Icon-FES) [50], mood will be assessed with the 15 item Geriatric Depression Score [39] and quality of life will be assessed using EQ-5D-5L [34].

#### Parkinson’s disease symptoms

Parkinson’s disease symptoms and stage will be assessed using the new Unified Parkinson’s Disease Rating Scale [44] (UPDRS) which incorporates a patient completed non motor and motor aspects of daily living, a clinician completed assessment of motor complications and results of a motor examination.

#### Falls

Falls and resultant injuries will be assessed as the number and rate of falls, proportion of fallers in each group, injury and injury related healthcare use. A fall will be defined as “an unexpected event in which participants come to rest on the ground, floor, or lower level” [51]. Falls will be recorded with falls diaries. All participants will receive monthly calendars on entry to the study which are returned in pre-paid envelopes to the research personnel. The calendars record the number of falls in a grid calendar and separate sheets record the circumstances for each fall. Participants will also be routinely telephoned monthly to verify any falls data and be screened for other adverse events.

#### Safety

Participants will be asked to notify the research team by phone if they experience any adverse events. Additionally, all participants will be screened during the monthly phone calls for adverse events using an open ended, non-leading question. Serious adverse events will be defined as events that result in death, hospitalisation (except for a pre-existing condition that had not worsened), significant disability or incapacity.

Potential worsening of extrapyramidal symptoms has been previously cited as a concern when prescribing cholinesterase inhibitors in Parkinson’s disease. A large multicentre placebo controlled trial of 541 people with mild to moderate dementia related to Parkinson’s disease demonstrated improvement in cognitive scores [45]. Worsening of tremor is a recognised side effect of rivastigmine but was only sufficiently severe to cause withdrawal of 1.7% of patients treated in the aforementioned trial. Monthly phone calls and access to the trial team should facilitate rapid reporting of such symptoms and appropriate down titration or cessation of the drug where clinically appropriate.

Adherence will be measured with a pill count of returned medications. This will be compared with the participant’s reported adherence on the basis of information ascertained during the follow-up phone calls and at the second face-to-face assessment visit.

### Sample size

As there are no data on the effect of rivastigmine treatment on step time variability, our sample size calculation has been estimated by extrapolating from related studies. For example, it has been found that treatment with donepezil resulted in a reduction in gait variability (22.3 to 11.3% CoV) for AD patients in a non-randomized trial [25].

Pragmatically, we estimate that we can recruit 130 eligible participants for the study over a 12 month period. Assuming a 30% drop out (based on a previous RCT [45]) this would result in approximately 90 patients (45 per arm) with trial data. This will enable us to detect a treatment effect difference of 0.6 standardised (z-score) units for our primary outcome measure (step time variability) with 80% power and at a two-sided 5% significance level. This sample size is similar to a study currently being undertaken in non-PD patients with mild cognitive impairment, i.e. 140 participants [28]. The use of a dual-task paradigm, as a cognitive stressor, may produce a more sensitive measure of any treatment benefit.

The clinical impact of such a group difference in gait variability can be estimated from a study by Maki [52]. In a cohort of 75 elderly volunteers without PD, a 0.6 standardised difference in stride velocity and stride length was associated with a 1.65 and 1.50 increased odds ratio of falling respectively, which would translate into clinically important benefits. We have assumed that these results will generalize to PD populations, and also manifest in step time variability, possibly a better predictor of fall risk in this population [53].

### Statistical analysis

Simple descriptive statistical analyses will initially be undertaken on all outcomes measures. For continuous measures (e.g. step time, step length and variability) the mean, SDs and distributions will be examined. Where measures are skewed, transformation will be undertaken. Outcome data on falls will be analysed by: (i) number of falls (count), (ii) non-fallers, low frequency of falls and frequent fallers (categorical ordinal variable), (iii) fall rate per person month (rate) and (iv) proportion of fallers in each group. Injury data will be summarized as (i) peripheral fracture rate per person-year of follow-up, (ii) number of peripheral fractures, (iii) number of people sustaining peripheral fractures, and (iv) number of people sustaining multiple events. For these variables, frequency counts (e.g. categorical variables) or Kaplan Meier plots for survival time variables will be analysed. Other binary or categorical measures (such as adverse event data) will be examined by simple frequency counts and contingency tables with Chi^2^ tests of association.

The main analyses will be performed using the intention to treat principle. For the primary outcome measure (step variability), we will use multivariable linear regression models and adjust for baseline or other covariates to increase precision. Hence we will compare group differences at 32 weeks in the gait variability parameters conditional on baseline parameters. For the secondary outcomes we can use ordinal logistic regression (e.g. for frequency of falls as an ordinal variable), Cox’s proportional hazard model for time to first fall and negative binomial regression to compare fall rates between groups. The last method accounts for falls clustering within participants, and has been recommended for falls data analysis [54]. The baseline covariates will be adjusted for as appropriate.

For participants who fail to complete the follow-up, sensitivity analyses will be undertaken, using multiple imputation methods. There are no planned interim analyses and no formal adjustments will be used for multiple testing though we will be cautious in our interpretation of the secondary outcomes (especially when the p-value is in the range of between 0.01 and 0.05) given the possibility of type I errors.

## Discussion

Falls and the consequences thereof are devastating to people with Parkinson’s disease. This trial will provide new insight into the effect of cholinesterase inhibitors on gait, balance and fall risk in this vulnerable group. It will establish and further validate falls prediction algorithms and delineate the interaction of multiple key risk factors that contribute to the high rate of falls that occur in this vulnerable population. Rivastigmine could potentially represent a safe and cost effective therapeutic approach to treating gait and balance dysfunction in Parkinson’s disease utilising an already established pharmacological agent. Hence, this may offer an effective, acceptable intervention in an area of Parkinson’s disease where there are currently unmet symptom needs and little in the way of evidence based therapeutic strategies.

## Appendix 1

The gait parameter definitions are described in Table 2. Step time variability is the primary outcome measure. The effect of the intervention will be measured on the other gait parameters listed.

**Table 2:** Gait parameter definitions used in the ReSPonD trial

**Gait parameter**	**Definition**
Stride	One stride equals two steps and represents one gait cycle.
Stride time	Time taken for one stride
Stride length	Distance traversed by two steps
Step	Defined by consecutive heels strikes of contralateral limbs
Step time	Time taken for one step
Step length	Distance between consecutive heel strikes, mean is calculated by the distance travelled / number of steps taken.
Walking speed	The velocity of walking, calculated as the distance travelled / time taken.
Variability	Variability of the measure of interest, expressed as the standard deviation (SD) and the coefficient of variation (CoV) = SD/mean x 100%.
Double limb support	The period for which both feet are in contact with the floor, calculated by time from heel strike until toe off of the contralateral limb.
Cadence (steps/min),	The number of steps per minute
Stance time (sec)	Duration of stance time (single and double support) in one gait cycle.
Walk ratio	The relationship between the amplitude and frequency of the rhythmic leg movements when walking. Calculated as step length / cadence.
Harmonic ratios (AP and VT)	The ratio of even/odd harmonics of acceleration over one gait cycle. In gait, AP and VT movements repeat each step. Stable gait therefore contains more AP and VT accelerations that repeat twice per stride as measured by even harmonics in the frequency domain.
Harmonic ratios (Matthew Brodie, personal communication, October 24th 2013)	The ratio of even/odd harmonics of acceleration over two gait cycles. In gait, ML movements repeat each stride. Stable gait therefore contains more ML accelerations that repeat twice every two gait cycles as measured by even harmonics in the frequency domain when two strides define the fundamental harmonic.

## Competing interests

SL: The Physiological Profile Assessment (NeuRA FallScreen) is commercially available through Neuroscience Research Australia.

No other competing interests declared.

## Authors’ contributions

AW and EH conceived the idea for the study. AW, YBS, EH, JC, SL and AL participated in the design of the study. EH drafted this manuscript for submission. All authors read and approved the final manuscript.

## Pre-publication history

The pre-publication history for this paper can be accessed here:

http://www.biomedcentral.com/1471-2377/13/188/prepub
